# Smartphone digital phenotyping, surveys, and cognitive assessments for global mental health: Initial data and clinical correlations from an international first episode psychosis study

**DOI:** 10.1177/20552076221133758

**Published:** 2022-11-08

**Authors:** Tanvi Lakhtakia, Ameya Bondre, Prabhat Kumar Chand, Nirmal Chaturvedi, Soumya Choudhary, Danielle Currey, Siddharth Dutt, Azaz Khan, Mohit Kumar, Snehil Gupta, Srilakshmi Nagendra, Preethi V Reddy, Abhijit Rozatkar, Luke Scheuer, Yogendra Sen, Ritu Shrivastava, Rahul Singh, Jagadisha Thirthalli, Deepak Kumar Tugnawat, Anant Bhan, John A Naslund, Vikram Patel, Matcheri Keshavan, Urvakhsh Meherwan Mehta, John Torous

**Affiliations:** 1 1859 Division of Digital Psychiatry, Beth Israel Deaconess Medical Center, Boston, Massachusetts, USA; 2 Sangath, Bhopal, India; 3 Department of Psychiatry, National Institute of Mental Health and Neurosciences, Bengaluru, Karnataka, India; 4 Department of Psychiatry, 390706 AIIMS Bhopal, All India Institute of Medical Sciences Bhopal, Bhopal, India; 5 Department of Global Health and Social Medicine, Harvard Medical School, Boston, Massachusetts, USA; 6 Department of Global Health and Population, Harvard T.H. Chan School of Public Health, Boston, Massachusetts, USA

**Keywords:** Schizophrenia, global health, digital health, smartphone apps, mental health

## Abstract

**Objective:**

To examine feasibility and acceptability of smartphone mental health app use for symptom, cognitive, and digital phenotyping monitoring among people with schizophrenia in India and the United States.

**Methods:**

Participants in Boston, USA and Bhopal and Bangalore, India used a smartphone app to monitor symptoms, play cognitive games, access relaxation and psychoeducation resources and for one month, with an initial clinical and cognitive assessment and a one-month follow-up clinical assessment. Engagement with the app was compared between study sites, by clinical symptom severity and by cognitive functioning. Digital phenotyping data collection was also compared between three sites.

**Results:**

By Kruskal-Wallis rank-sum test, we found no difference between app activities completed or digital phenotyping data collected across the three study sites. App use also did not correlate to clinical or cognitive assessment scores. When using the app for symptom monitoring, preliminary findings suggest app-based assessment correlate with standard cognitive and clinical assessments.

**Conclusions:**

Smartphone app for symptom monitoring and digital phenotyping for individuals with schizophrenia appears feasible and acceptable in a global context. Clinical utility of this app for real-time assessments is promising, but further research is necessary to determine the long-term efficacy and generalizability for serious mental illness.

## Introduction

Mental health conditions like schizophrenia impact ∼1% of the global population. Yet access to effective care for people with schizophrenia remains limited in all countries and especially in low- and middle-income countries.^1,2^ Given that early interventions can be effective and change the lifelong trajectory of the illness,^3^ efforts to provide scalable and accessible care for schizophrenia are a global mental health priority. Digital health technologies, especially smartphones represent one promising approach toward offering such scalable and accessible care.

Smartphone ownership in both high and low-middle income countries, with reported rates around 90% in the United States^4^ and around 45% in India.^5^ Smartphone use specifically among individuals with schizophrenia with rates is also on the rise, between 50 and 60% in the United States.^6,7^ Numerous studies have shown that youth with schizophrenia are willing and interested in using their smartphone as a tool for recovery,^8–10^ though some barriers for older adults to engage remain.^11^ Research has also shown that smartphones can effectively deliver evidence-based resources (e.g., psychoeducation and therapy skills) to people with schizophrenia spectrum disorders.^12^ Parallel research has also shown that smartphones can be used to monitor recovery and risk of relapse through digital phenotyping methods—using passive data captures through multimodal sensors built into smartphones to assess real-time and longitudinal trends around symptoms, sleep, physical activity, etc.^13,14^ Taken together the digital phenotyping potential, app intervention delivery, and already high access to smartphones makes them an ideal tool to bridge the gap in care for schizophrenia.

One potentially promising use case for apps is around relapse prevention, using digital phenotyping data to predict relapse and digital interventions to offer tailored support in response to early warning signs detected through real-time analysis of multi-modal data streams captured through a smartphone app.^15^ While this is the focus of our team's work, a first step in building better models to predict relapse is to understand the nature of the data gathered and its clinical meaning.^16^ The digital phenotyping data streams that are a focus of this paper include what is often referred to as active data (surveys on the smartphone filled by the user), passive data (geolocation, screen-time, anonymized call/text logs, and accelerometer), cognitive data (from user assessments in the apps), and meta-data related to engagement with the app itself. While an increasing number of studies are now capturing this data in schizophrenia research ,^17^ such app-based efforts are often tailored to one site or one team, and the feasibility and acceptability of capturing this type of data across a range of high and lower income settings remain unknown. In the context of this paper, we define feasibility and acceptability in the context of generating clinically relevant and research-grade data across the first month of app use at all three sites.

Yet, most research and apps related to schizophrenia have not realized this scalable potential. Numerous apps have already been created and each offers interesting results and use cases in both high and low-middle income countries.^18,19^ But until apps can be broadly disseminated and used across diverse settings and cultures, their impact will remain limited.^20^ Thus, the focus of this paper is to explore the use of an app to analyze clinical and functional data of individuals with schizophrenia across one site in the United States and two in India. Following our published protocol,^21^ here we report on the initial experience over the first month around patient use of the app and data collection. Based on our prior research and co-design of the app with patients living with schizophrenia spectrum disorders, their family members, and clinicians,^22^ we hypothesized that patients at all sites would engage with the app at or above the suggested study metrics for activity completion. We also hypothesized that digital phenotyping data related to sensors (e.g., location) would be similar across all study sites and reflect the common biological underpinnings of the illness irrespective of site or culture. Likewise, we also hypothesized that patients would report core symptoms of their illness in a similar manner across all study sites, again reflecting the shared experience of schizophrenia as a biological illness.

## Methods

All research protocols and data sharing were approved by Institutional Review Boards at each site. Written consent was obtained from all participants.

### Recruitment

Participants were recruited by Beth Israel Deaconess Medical Center (BIDMC) in Boston, USA, by the National Institute for Mental Health and Neuro-Sciences (NIMHANS) in Bengaluru, India, by Sangath in Bhopal, India in collaboration with the All India Institute for Medical Sciences (Sangath-AIIMS Bhopal), beginning in September 2021. The inclusion criteria for all participants were a diagnosis of a psychotic spectrum disorder or a report of symptom onset for psychosis, within the 5 years prior to the beginning of data collection in 2021, and less than 45 years in age. At BIDMC, this was ascertained by the date of diagnosis and the age of the participant, as reported by the participant, and at NIMHANS and Sangath-AIIMS Bhopal this was ascertained by medical records. At BIDMC, additional inclusion criteria were that participants lived anywhere in the US, had English proficiency, and possessed a smartphone. The inclusion criteria at NIMHANS and Sangath-AIIMS were that participants lived in India and had sufficient English or Kannada proficiency (in Bengaluru, Kartnataka) or sufficient English or Hindi proficiency (in Bhopal, Madhya Pradesh). Participants at BIDMC were recruited online via ResearchMatch and Craigslist, by referrals from BIDMC clinicians, or by flyers distributed and posted at BIDMC and at the Massachusetts Mental Health Center in Boston. Participants at NIMHANS were recruited from the outpatient services within the hospital, and participants at Sangath-AIIMS Bhopal were recruited at the All India Institute of Medical Sciences outpatient psychiatry services in Bhopal.

### Protocol

Each participant had an initial intake visit and a one-month follow-up visit at each site. Participants met with research staff initially by video call at BIDMC or in-person at NIMHANS and Sangath-AIIMS Bhopal. During the initial visit, participants were given a detailed description of the study and walked through the informed consent form, with the opportunity to ask questions about the study and to ensure full understanding of what participation in this study involves. Once written consent was obtained, participants were then guided to download the smartphone app, mindLAMP 2, from the App Store on Apple devices or the Play Store for Android Devices, and a walkthrough of the app and the necessary settings were provided by the researcher. For participants at the India sites who did not have a smartphone or whose smartphone only supported software older than Android 8 or iOS 11 (which could not support mindLAMP), a Samsung Galaxy M31, chosen for its suitability for real-time data capture after testing against 11 smartphone models, was provided for the duration of the study along with a mobile data plan where necessary. Before the start of the study, a one-week trial period was included to observe and ensure sufficient passive data collection via the smartphone, which helped ensure those using their own smartphone could meaningfully partake in the study.

Following the app setup, clinical symptoms for psychosis were assessed by the Positive and Negative Syndrome Scale (PANSS)^23^ and cognitive symptoms were assessed by the Brief Assessment of Cognition Scale (BACS),^24^ for which inter-rater reliability training between the researchers at all three sites was done. At BIDMC, the Token Motor task or the Symbol Coding task were not administered at the first visit due to the limitation of virtual administration. Functioning and other clinical symptoms were assessed by the following self-report scales: Patient Health Questionnaire (PHQ-9)^25^ Generalized Anxiety Disorder (GAD-7),^26^ Social Functioning Scale,^27^ Short Form Health Survey,^28^ Behavior and Symptom Identification Scale 24 (BASIS-24),^29^ Warning Signal Scale (WSS),^30^ the Pittsburgh Sleep Quality Index (PSQI).^31^ A Clinical Global Impression Scale (CGI)^32^ was completed by the clinician, and participants were also asked to report if they contracted COVID-19. At BIDMC, these were collected via the online survey platform REDCap ,^33,34^ and at NIMHANS and Sangath-AIIMS Bhopal these were collected on-paper by self-administered forms. Monthly follow-up visits were conducted via remote videoconferencing at BIDMC, in-person at Sangath-AIIMS Bhopal, and either by telephone or in-person by NIMHANS based on participant preference, and depending on the latest COVID-19 safety guidelines implemented at each of the study sites. All assessments were repeated at the one-month follow-up visit, except the BACS.

Participants at BIDMC received $20 at each visit, with additional payments of $30 for initial, 6-month, and final visits. Participants who received the wrist actigraph also received an additional $50 upon its return. Participants at NIMHANS and Sangath were to be compensated with INR 500 (∼$7) for each of their visits. Beyond the first month of data analyzed here, all follow-up visits follow the same protocol as the first month, with only the BACS additionally administered at the 6-month and 12-month visits.

### mindLAMP protocol

Over the 12 months, participants received daily notifications for app-based activities on the mindLAMP app, including ecological momentary assessments (EMA) surveys, cognitive games, psychoeducation resources, and relaxation exercises. Participants could see the scheduled activities on the Feed page of the app, which appeared as a daily to-do list Participants were encouraged to complete activities four times per week, and researchers would communicate with participants regarding engagement with the app, and provide technical support when necessary and if they did not use the app for over five days.

### Symptom monitoring: EMA surveys & cognitive games

Mood, anxiety, psychosis, sleep, social functioning, and medication adherence (EMA) surveys were randomized such that two of six were sent to users twice per day, to be completed once. These surveys aimed to reduce recall bias and increase the ecological validity of the findings while complementing the monthly follow-up assessments.^35^ The mood and anxiety surveys matched the PHQ-9 and GAD-7 exactly, whereas the sleep, social, and psychosis surveys were adapted from the Social Functioning Scale, PSQI, and PANSS. The cognitive games available on the app were Jewels A and B (an adaptation of Trail Making Test A and B^36^), Box Game (backwards spatial span), Cats and Dogs (spatial span), Balloon Risk (a risk-reward task), Pop the Bubbles (a go/no-go task). These were randomized such that two of six were sent to users once per day.

### Interventions: psychoeducation resources & relaxation exercises

Psychoeducation resources, called “learn tips” within the app, were symptom and lifestyle management skills adapted from various online resources for people with schizophrenia. Relaxation activities within the app include guided sessions that participants could follow along with, of various lengths and types. These resources were available to participants for use at their discretion. Participants received two default notifications for relaxation activities per week, and an adaptive notification schedule was implemented such that when scores on the EMA surveys increased, a relevant learn tip or relaxation activity was sent to the participant—unlike the other notifications, however, this did not appear on the Feed page, was not scheduled at regular times, and was not encouraged or discouraged by research staff, unlike the symptom monitoring activities.

### Analysis

This analysis focuses only on the first month of data collected from the ongoing SHARP study including data collected from the app and at the initial and first follow-up visits. We conducted between-site comparisons of participant demographics, symptom severity, mindLAMP engagement, passive data quality, EMA-reported symptoms as they varied by clinically assessed symptom severity, and cognitive game scores as they varied by clinically-assessed cognition. To limit the scope of these analyses, we included only psychosis-related clinical and cognitive functioning measured by PANSS and BACS. Further, mood, anxiety, sleep, and early signs of relapse were analyzed with the PHQ-9, GAD-7, PSQI, and WSS, respectively. We included only analysis on the Jewels A and B task as these are the first to be validated against paper-and-pencil versions of the task.^37^

Demographic information regarding age, gender, race, and ethnicity was taken from additional questions on the BASIS-24. Religious identity was additionally reported due to its saliency over more homogenous categories of race and ethnicity. Each domain on the BACS was scaled in accordance with the task guidelines.^38^ To accommodate COVID-related limitations on data collection, the scaled score for the four domains collected at each site was averaged and reported as the BACS Combined Scaled Score, and the scaled Symbol Coding score and Token Motor score were collected at the two India sites were reported separately. Demographic and clinical symptom analysis included all participants who completed the initial visit. Subsequent analyses only included participants who completed the one-month follow-up visit and who had mindLAMP data.

App engagement was measured by the average number of activities completed per day over a 30-day period. GPS data quality was derived from the number of data points collected compared to expected data points based on the pre-determined sampling rate. We used 50% as a threshold for good quality data. When comparing symptom severity between clinical assessment and the EMA surveys, all scores were standardized on a zero to one scale. The two Jewels games were scored by average time between taps in a single Jewels session, where a tap was only counted if it was on the correct jewel in the sequence. Outliers who completed >1000 activities (more than 30 a day) were removed from engagement-related analyses, and outliers who took longer than 5 s per tap on average on either Jewels task were excluded.

Preprocessing pipelines for mindLAMP activities and passive data were developed in-house at BIDMC (LAMP Consortium and Division of Digital Psychiatry at BIDMC). mindLAMP data for the India sites was preprocessed at Bengaluru, in compliance with the data use agreement stipulated by the NIMHANS and Sangath-AIIMS Bhopal IRB, and only de-identified data were supplied for analysis. All pre-processing was completed in Python. All data from the monthly visits were preprocessed at BIDMC, with de-identified data supplied by each site in accordance with NIMHANS and Sangath IRB and AIIMS Bhopal IHEC. All analyses and visualizations were completed using R packages “gt,” “gtsummary,” “ggpubr,” “ggpmisc,” and “flextable.” Fisher's exact test was used for categorical demographic variables, and the Kruskal-Wallis rank-sum test was used for comparisons between all three sites on clinical assessments, followed by the Wilcoxon rank-sum test for non-parametric data for analysis between pairs of sites, with 5% level of significance in each case. Finally, linear regressions were used to study the relationship between clinical measures and mindLAMP data.

## Results

### Demographics and clinical symptom severity

60 participants completed the initial visit (17 at BIDMC, 20 at NIMHANS, and 23 at Sangath-AIIMS Bhopal). No significant differences were found between the age and gender of participants at each site. Expectedly, the BIDMC participants differed by race and ethnicity from NIMHANS and Sangath-AIIMS Bhopal participants. Table 1 details the participants’ demographics at each of the three sites. No significant difference was found between participants’ religion between the two Indian sites, details of which are described in Table 2.

**Table 1.** Participant demographics in total and across all three sites.

Demographic	Overall, N = 60^a^	BIDMC, N = 17^a^	NIMHANS, N = 20^a^	Sangath-AIIMS Bhopal, N = 23^a^	p-value
Age	30.62 (7.25)	30.59 (8.11)	32.40 (7.04)	29.09 (6.70)	0.2^b^
Sex					0.068^c^
Female	30 (50%)	12 (71%)	8 (40%)	10 (43%)	
Male	29 (48%)	4 (24%)	12 (60%)	13 (57%)	
Non-binary	1 (1.7%)	1 (5.9%)	0 (0%)	0 (0%)	
Ethnicity					0.001^c^
Hispanic or Latino	5 (8.3%)	5 (29%)	0 (0%)	0 (0%)	
NOT Hispanic or Latino	55 (92%)	12 (71%)	20 (100%)	23 (100%)	
Race					<0.001^c^
African American	4 (6.7%)	4 (24%)	0 (0%)	0 (0%)	
Asian	43 (72%)	0 (0%)	20 (100%)	23 (100%)	
Multiracial or other	2 (3.3%)	2 (12%)	0 (0%)	0 (0%)	
White	11 (18%)	11 (65%)	0 (0%)	0 (0%)	

^a^ Mean (SD); n (%).

^b^ Kruskal-Wallis rank-sum test

^c^ Fisher's exact test

**Table 2.** Participants’ demographics by religion, for India study sites.

Demographic	Overall, N = 43^a^	NIMHANS, N = 20^a^	Sangath-AIIMS Bhopal, N = 23^a^	p-value^b^
Religion				0.7
Christian	1 (2.3%)	1 (5.0%)	0 (0%)	
Hindu	40 (93%)	18 (90%)	22 (96%)	
Muslim	2 (4.7%)	1 (5.0%)	1 (4.3%)	

^a^ n (%).

^b^ Fisher's exact test between sites.

Overall, psychotic symptom severity was mild across the three sites with Sangath-AIIMS Bhopal participants having the highest mean PANSS total score of 55 (SD = 16), and NIMHANS participants having the lowest mean 37 (SD = 15). NIMHANS site overall had the least severe symptoms along PHQ-9, GAD-7, WSS, and PSQI as well, which were significantly different between the sites on all self-report assessments and all PANSS subdomains. Cognitive functioning measured by the BACS was highest among participants at BIDMC lowest for participants at Sangath-AIIMS Bhopal on the four domains measured at all three sites, with significant differences between all three sites emerging on the digit sequencing task (p = 0.001), verbal fluency task (p < 0.001) and Tower of London tasks (p = 0.004), and between Sangath-AIIMS Bhopal and NIMHANS on the symbol coding task (p = 0.004). Details regarding the mean, standard deviation, and p-value for each of the study sites on all clinical assessments are outlined in Table 3. No serious adverse events were reported by participants at any site.

**Table 3.** Mean scores of the participants on the clinical assessment at baseline for each study site.

Assessments		Overall, N = 60^a^	BIDMC, N = 17^a^	NIMHANS, N = 20^a^	Sangath-AIIMS Bhopal, N = 23^a^	p-value^b^
PANSS	Positive	12 (5)	16 (5)	8 (2)	13 (5)	<0.001
	Negative	11 (4)	10 (3)	10 (3)	13 (5)	0.039
	General	25 (7)	27 (4)	19 (3)	29 (8)	<0.001
	Total	49 (14)	53 (7)	37 (5)	55 (16)	<0.001
Self-Report	PHQ-9	11 (8)	14 (6)	3 (4)	17 (5)	<0.001
	GAD-7	8 (7)	10 (6)	2 (3)	13 (5)	<0.001
	WSS	6 (5)	5 (2)	1 (2)	12 (2)	<0.001
	PSQI	7 (5)	12 (5)	2 (2)	6 (3)	<0.001
BACS	Verbal Memory	−2.06 (1.33)	−1.42 (1.17)	−2.37 (1.23)	−2.27 (1.41)	0.088
	Digit Sequencing	−1.71 (1.53)	−0.97 (1.11)	−1.38 (1.47)	−2.56 (1.50)	0.003
	Verbal Fluency	−1.56 (1.42)	−0.54 (1.31)	−1.16 (0.83)	−2.67 (1.16)	<0.001
	Tower of London	−1.63 (2.17)	−0.89 (1.93)	−1.19 (2.09)	−2.56 (2.16)	0.005
	Symbol Coding	−2.36 (1.39)	NA (NA)	−1.82 (1.23)	−2.82 (1.38)	0.009
	Token Motor Task	−2.28 (1.17)	NA (NA)	−2.06 (1.03)	−2.48 (1.26)	0.2

^a^ Mean (SD).

^b^ Kruskal-Wallis rank-sum test.

Of the 60 total participants who completed the initial visit, 53 also completed the first-month follow-up and had mindLAMP data (13 at BIDMC, 18 at NIMHANS, 22 at Sangath). Subsequent analyses include only these 53 participants. Details regarding the demographics and clinical profiles of the subset of completers, and between pairs of sites can be found in Appendix 1–4.

### Engagement

**Figure 1.**
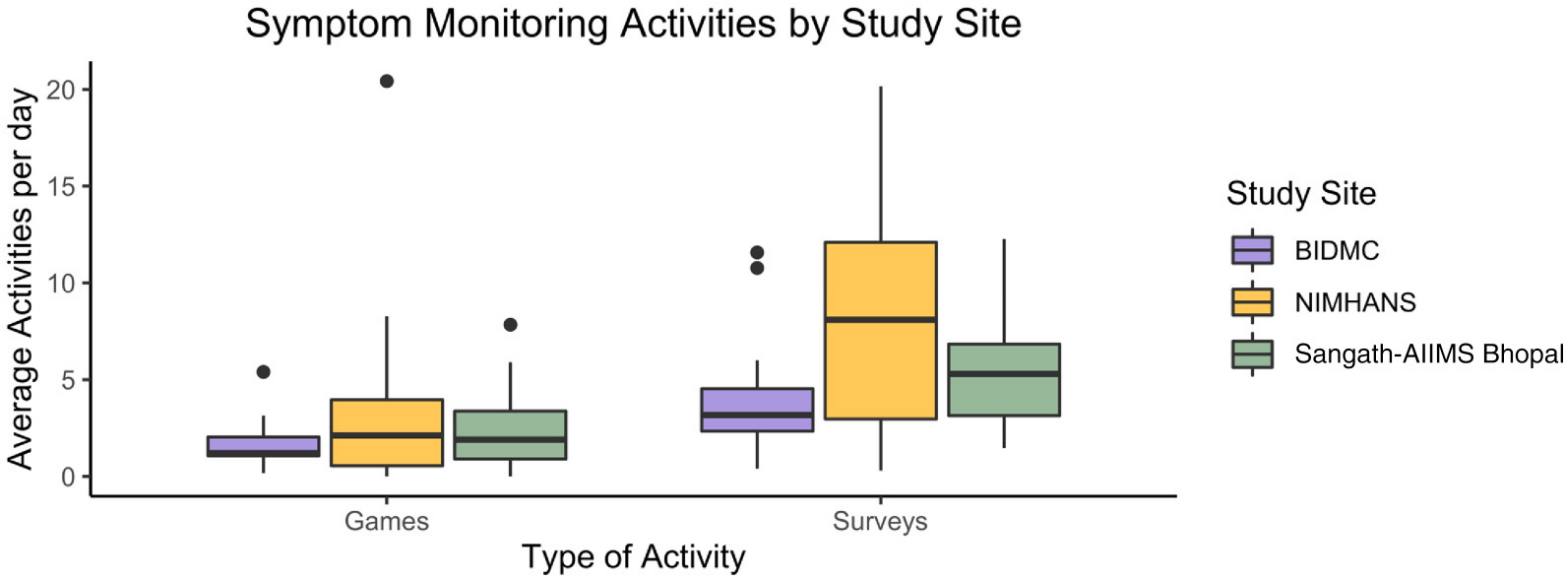
Mean games and surveys were completed per day by participants at each site. Participants received notifications to complete surveys twice per day and to complete games once per day.

The total number of activities completed per day was 8.8 (7.0) with no significant difference in overall mean completion across sites. The mean survey activities (5.9 (4.2)) and games (2.58 (3.22)) per day were also not significantly different across the three sites. The distribution of these symptom monitoring activities is reported in Figure 1. The mean of tips (4.0 (10.7)) and relaxation activities (5 (11)) completed per month were significantly different between the study sites (p = 0.015; p = 0.025) with BIDMC participants completing the most relaxation (6 (7)) and Sangath participants completing the most tips (5.9 (9.6)) per month (see Appendix 5-6).

**Figure 2.**
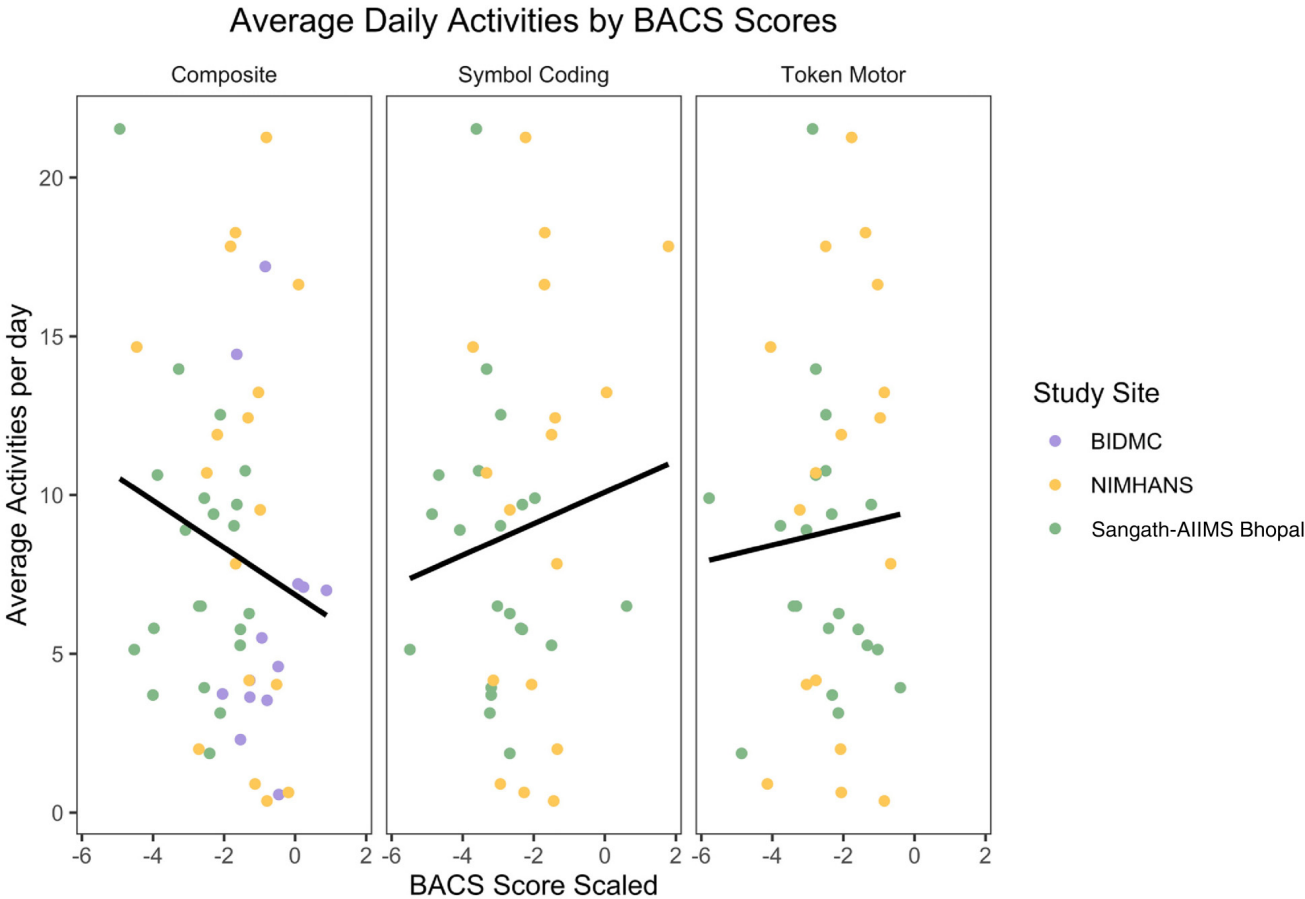
Mean activities completed per day by BACS composite scaled score, symbol coding score, and token motor score.

**Figure 3.**
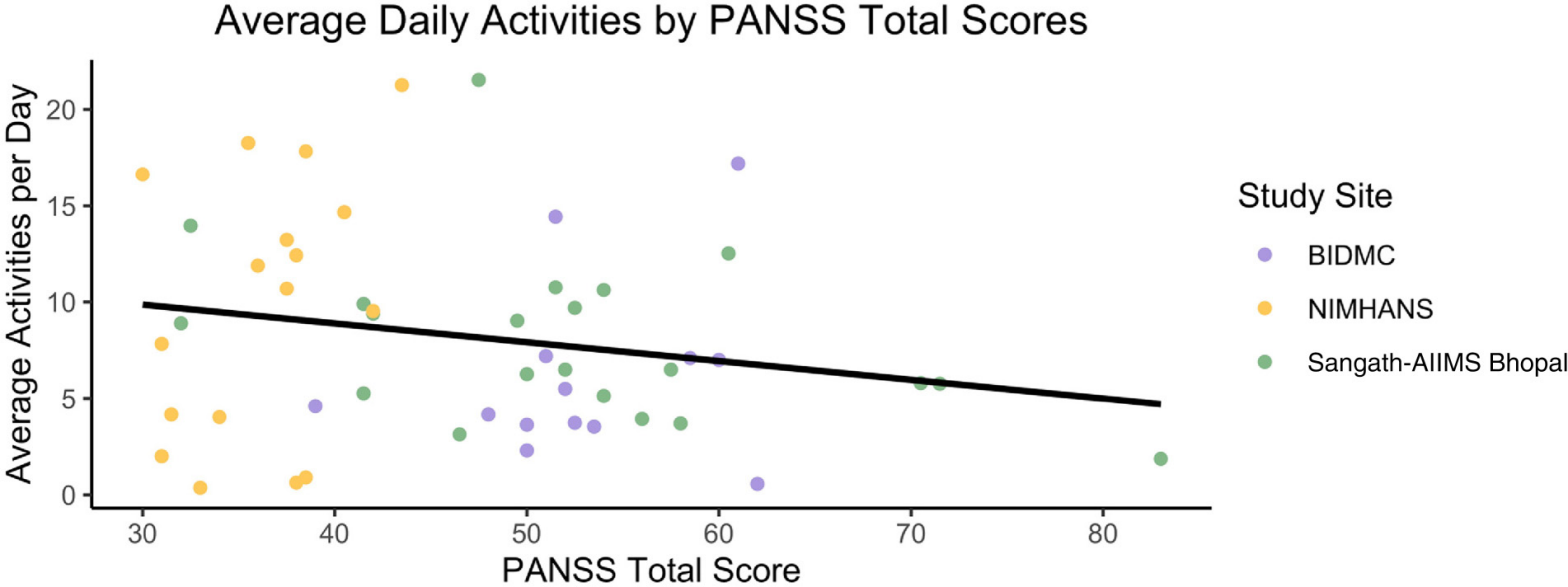
Mean activities completed per day by total PANSS score.

No significant difference was found between the BACS domains and the average number of surveys and games completed per day (Figure 2), or between symptom severity on PANSS and activity completion (Figure 3).

### Passive data quality

**Figure 4.**
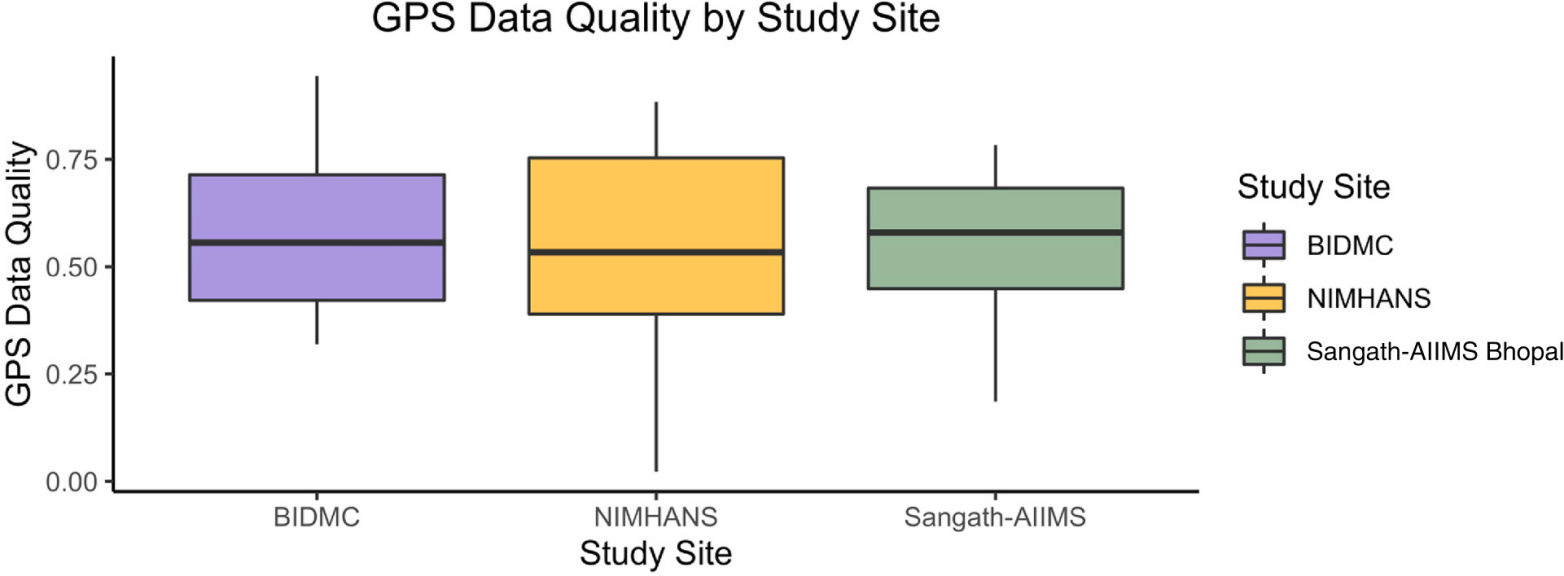
Boxplots showing the quality of GPS data collected at each study site.

No significant difference emerged between GPS data quality between the three study sites, and all sites had mean GPS data quality greater than 50%. Figure 4 shows the distribution of GPS data quality collected at each site.

Examining digital phenotyping based on passive data, we were able to generate a feature called “home time” and a feature called “entropy” to describe participants’ day-to-day movements. A person who stays home all week would have low entropy whereas a person who visits new locations each day and has no routines in their mobility would have higher entropy. We did find a significant difference between home time (p = 0.038) with BIDMC being the lowest mean at 13.2 (6.7) and NIMHANS being the highest at 20.3 (1.8) (see Appendix 7-8).

### mindLAMP real-time self-report

**Figure 5.**
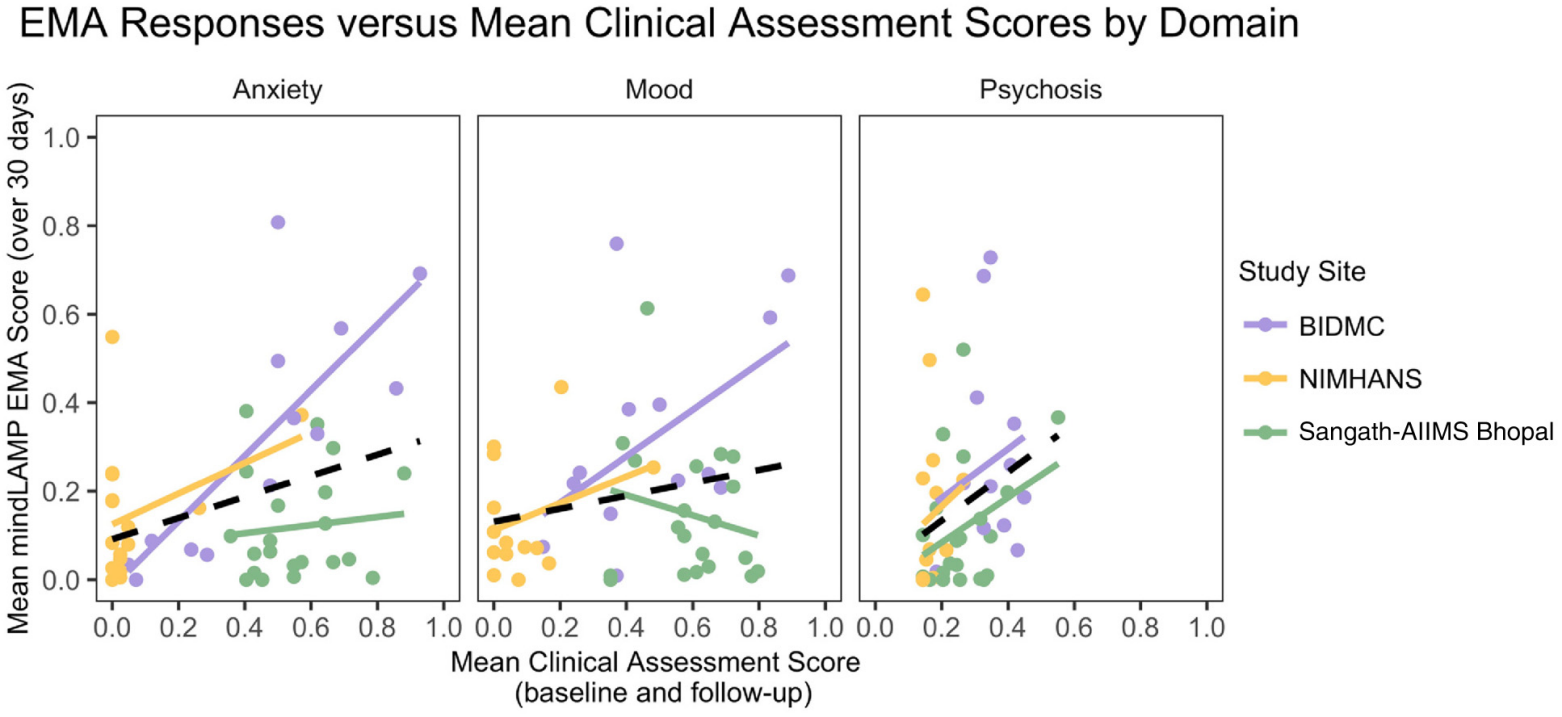
Mean EMA scores over 30 days versus average clinical assessment scores, between baseline and follow-up, for each study site and in total, by anxiety, mood, and psychosis symptom domains. Mean scores captured via EMA.

We analyzed domain-specific relationships between mean clinical assessments at baseline and follow-up and mean daily EMA surveys on mindLAMP over 30 days, hypothesizing a linear association between EMA surveys and clinical assessment scores. Figure 5 details the relation between mean GAD-7, PHQ-9, and PANSS Positive scores, standardized on a zero to one scale, and the mean response to anxiety, mood, and psychosis surveys administered daily on mindLAMP over the month.

Overall, symptoms reported on the EMA surveys were correlated with clinical assessments for anxiety (R^2^ = 0.125, p = 0.012) and psychosis (R^2^ = 0.085, p = 0.04), though these do not explain much of the variance in EMA responses. Examining this further by each study site, only the anxiety EMA responses were strongly predicted by clinical assessment at BIDMC, with the model explaining 63% of the variance (R^2^ = 0.626, p = 0.001). The relation between clinical assessments and EMA responses for each site and each domain is detailed in Figure 5.

**Figure 6.**
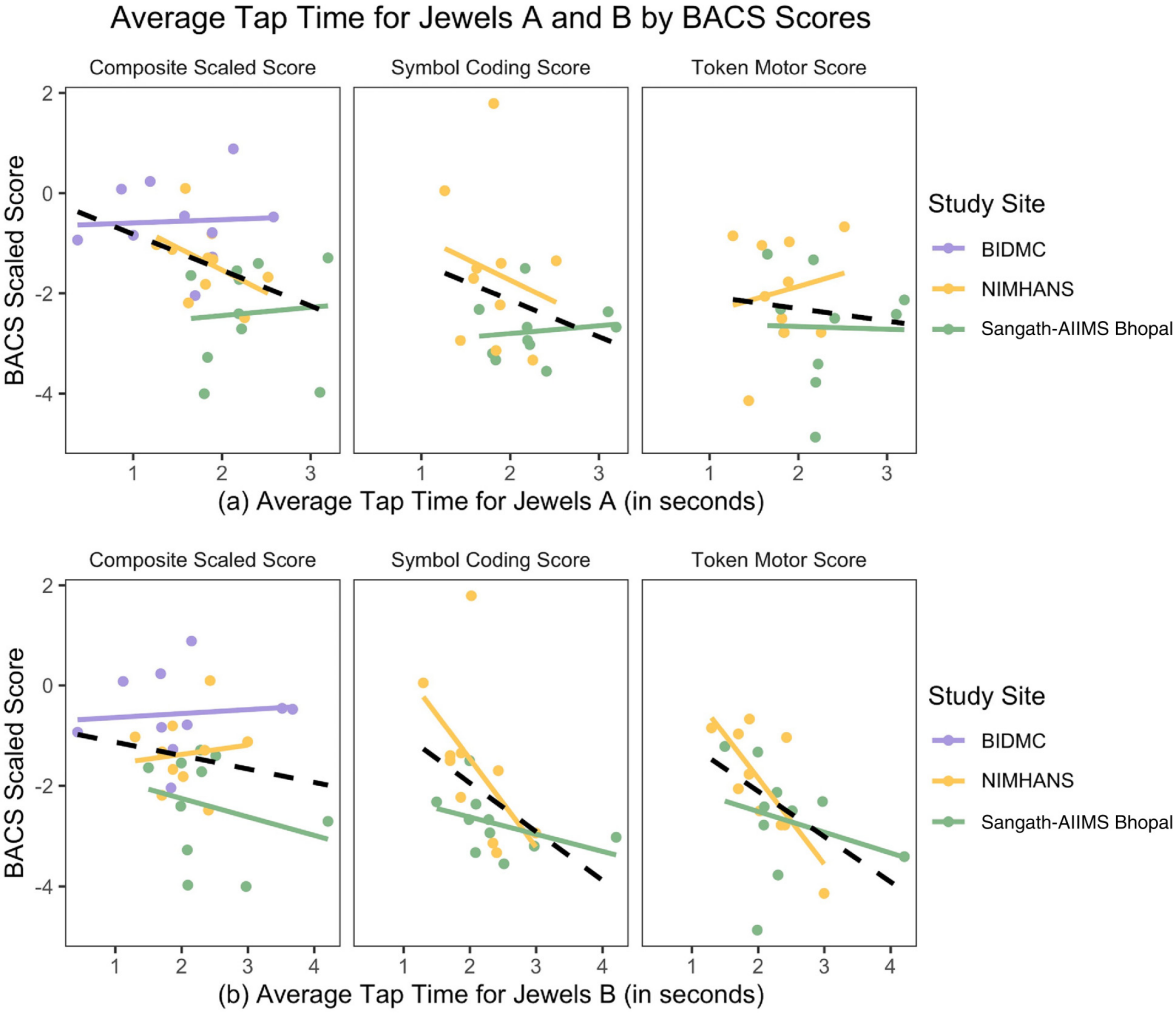
Average tap time for (a) jewels A and (b) jewels b (in seconds) for each study site, as varied by BACS composite scaled score, symbol coding score, and token motor score.

In exploring the relation between cognition scores on the BACS and performance on the Jewels games, we found that higher cognitive scores appeared to correlate with quicker responses on both Jewels tasks. For Jewels A (Figure 6(a)), the BACS composite score significantly predicted average tap time (R^2^ = 0.142, p = 0.040). For Jewels B (Figure 6(b)), symbol coding task (R^2^ = 0.223, p = 0.036) and the token motor task (R^2^ = 0.251, p = 0.025) both significantly predicted average tap time on the jewels. When examined by individual study site, no significant correlations emerged for Jewels A and only one, token motor score at NIMHANS was significant for Jewels B(R^2^ = 0.561, p = 0.013).

## Discussion

This study investigated 30 days of data collected from a smartphone-based observation and digital phenotyping study of individuals with early psychosis across different cultures and contexts in the United States and India. We found that overall participants completed 8.8 activities per day, with no difference in the number of activities by location (Figure 1). There was also no observed effect of illness severity, determined by BACS or PANSS scores on the number of activities completed. For participants across all three study sites, we were able to collect at least 50% of expected GPS data points on average. These findings suggest international research using the digital phenotyping methods is practical, both feasible and acceptable, and can generate novel data. With increasing access and use of smartphones across most settings globally, the use of digital phenotyping is also potentially scalable across different populations.

Our results suggest that the use of the same app across different cultures and countries with high engagement is feasible in conjunction with regular clinical care. This suggests the potential for leveraging digital applications to augment clinical care for mental disorders across diverse settings globally. The overall high number of activities completed is notable in light of low engagement in most health apps now being recognized as the chief limiting factor in their clinical implementation.^39^ Likely, co-designing the app with users,^22^ and regular communication with researchers helped with achieving this relatively high engagement, as our results show similar high engagement across participants from all three study sites. Engagement with the app did not appear to correlate with severity for psychosis symptoms or related cognitive functioning, suggesting that even patients with relatively higher symptom burdens were able to use the app. This engagement without reported adverse events suggests against stigmatizing claims that people with schizophrenia may be too paranoid to engage with apps. This contributes to the growing evidence across mental health research highlighting the importance of stakeholder-driven interventions in global mental health.^40^

As we assess the role of digital phenotyping to inform clinical care for schizophrenia, our results show promise in the feasibility and acceptability of leveraging a smartphone app for enabling passive data collection among a vulnerable patient population across diverse contexts. The consistent data quality at all three sites is especially important as this supports the technological feasibility of collecting passive data in rural and urban settings, and using it to generate phenotyping features such as entropy, which categorizes the variability in location per day, and home time which calculates hours spent in a home location per day (Appendix Figures 2 and 3). This remains an avenue for future research, in examining the clinical significance and intergroup differences of these features.

Emerging evidence from our results suggests there may be a significant relationship between clinically-assessed versus app-reported symptom severity, and clinically assessed cognitive performance with results from app-based cognitive assessments. The lack of a high correlation between clinical versus app derived metrics highlights the potential of digital phenotyping methods to capture more dynamic symptom profiles in the context of complex potential mediators or moderators like sleep, exercise, social interactions, etc. Our findings also call attention to the need for further research exploring possible factors affecting how people reported symptoms to an app and whether levels of trust in the researcher versus in the app, or self-perception of momentary versus month-long severity of symptoms, may explain the differences in symptom reporting.

Our findings on the use of Jewels cognitive assessment on the app provide early suggestions for how smartphone-based assessments could be developed to offer easily accessible screening data not otherwise available for routine care, given how challenging assessing cognition in schizophrenia is in routine clinical care and with the COVID-19 pandemic has made this even more so with face-to-face services limited. A 2022 scoping review of remote cognitive assessments for the field suggested few approaches have actually been deployed into truly remote settings, and more research is needed to explore this approach across different cultures and settings like in this present study.^41^

The limitations of this research were driven primarily by the impacts of COVID-19, though we highlight potential limitations in our sample population as well. This research was conducted during COVID-19 which placed new challenges on recruitment and also meant that results may or may not generalize outside of this unique period in time. The nature, severity, and response to COVID-19 were of course different in India and the United States, meaning that each site had different stressors that impacted recruitment and patients at different times from the others. For example, in Boston, the study was forced to be conducted entirely remotely, and while appropriate for a smartphone study, this did limit the ability to provide more hands-on support and build alliances with participants, and may also have impacted the population sample in terms of symptom severity and digital literacy. The differences in severity of illness across the sites coupled with the small sample size also make it challenging to make broad claims around clinical impact, although it does support this approach working across different clinical settings. Furthermore, all participants in this study had a diagnosis of a schizophrenia spectrum disorder, were engaged in care, and did not have severe symptoms. This limits the generalizability of our findings, and further highlights the need to consider how digital mental health applications could be leveraged for reaching patients not engaged in care, or who may be unable or reluctant to seek formal services. Given our focus on younger people with schizophrenia, we cannot say if these results may generalize to older people with the illness. All participants in this study were fluent in the primary local language at each site (i.e., Kannada at the NIMHANS site, and Hindi at the AIIMS-Sangath site); however, it is likely that some participants were also familiar with English. We did not specifically measure English language fluency or if they utilized the English or Hundi language version of the app, but it is possible that participants at the sites in India who could speak English may have differed on other characteristics, such as sociodemographic status, when compared to participants at those sites who could not speak English, which could introduce potential bias in our findings. Lastly, with only one month of data reported here, it is not possible to draw conclusions about the clinical patterns demonstrated in our data; our final study results will yield additional necessary insights, demonstrate the potential for longitudinal data capture through a digital phenotyping app, and inform necessary replication. Despite these limitations, our findings offer valuable initial insights into the feasibility and acceptability of a digital phenotyping app for supporting data capture among a population with schizophrenia spectrum disorders across diverse settings in the United States and India.

## Conclusions

Our findings support evidence that app-based tools for symptom monitoring and digital phenotyping among individuals with schizophrenia are feasible and acceptable in global contexts. These findings from the first month of data in an ongoing study suggest app engagement between sites in India and the United States was similar. Further explorations into the long-term engagement with, and clinical utility of the smartphone-based tool, are necessary, as early trends indicate the potential to capture real-time symptom severity and cognitive functioning, which may be crucial for advances in smartphone-based relapse prediction as an adjuvant to clinical care. Our study was limited by a small sample size and the impact of COVID-19. However, trends in smartphone use in India and the United States suggest that such a tool may be scalable to low-middle income countries to meet burgeoning needs for mental health services as an adjuvant to clinical care.

## Figures and Tables

**Appendix 5. fig7-20552076221133758:**
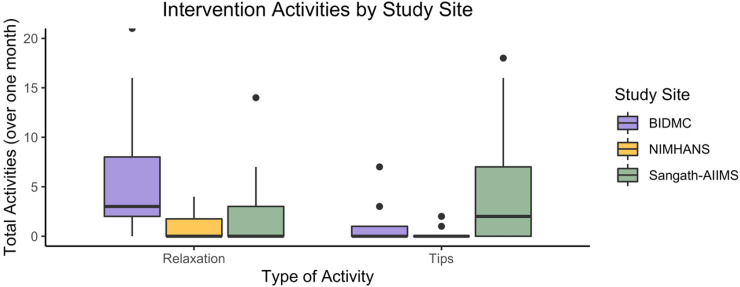
Relaxation and psychoeducation tips completed over one month by participants at each site. Participants received 2 relaxation notifications per week and periodic notifications about tips based on reported symptoms.

**Appendix 7. fig8-20552076221133758:**
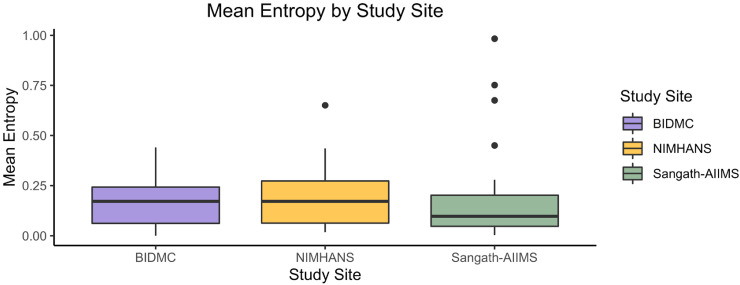
Mean entropy (representing variation in location in a day) of participants by study site, derived from location-based passive data.

**Appendix 8. fig9-20552076221133758:**
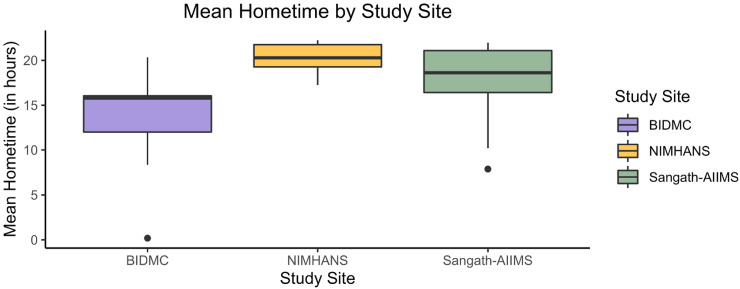
Time spent at home in hours for participants by study site, derived from location-based passive data.

## Appendices

[Table table4-20552076221133758]

**Appendix 1. table4-20552076221133758:** Baseline demographics and assessments between BIDMC and NIMHANS sites.

Variable	Overall, N = 37^a^	BIDMC, N = 17^a^	NIMHANS, N = 20^a^	p-value^b^
Age	31 (7)	31 (8)	32 (7)	0.2
Sex				0.068
Female	30 (50%)	12 (71%)	8 (40%)	
Male	29 (48%)	4 (24%)	12 (60%)	
Non-binary	1 (1.7%)	1 (5.9%)	0 (0%)	
Ethnicity				0.001
Hispanic or Latino	5 (8.3%)	5 (29%)	0 (0%)	
NOT Hispanic or Latino	55 (92%)	12 (71%)	20 (100%)	
Race				<0.001
African American	4 (6.7%)	4 (24%)	0 (0%)	
Asian	43 (72%)	0 (0%)	20 (100%)	
Multiracial or other	2 (3.3%)	2 (12%)	0 (0%)	
White	11 (18%)	11 (65%)	0 (0%)	
PHQ-9	8 (7)	14 (6)	3 (4)	<0.001
GAD-7	5 (6)	10 (6)	2 (3)	<0.001
WSS	3 (3)	5 (2)	1 (2)	<0.001
PSQI	7 (6)	12 (5)	2 (2)	<0.001
PANSS				
Positive	12 (5)	16 (5)	8 (2)	<0.001
Negative	10 (3)	10 (3)	10 (3)	0.6
General	23 (5)	27 (4)	19 (3)	<0.001
Total	44 (10)	53 (7)	37 (5)	<0.001
BACS				
Verbal Memory	−1.94 (1.28)	−1.42 (1.17)	−2.37 (1.23)	0.034
Digit Sequencing	−1.19 (1.31)	−0.97 (1.11)	−1.38 (1.47)	0.4
Verbal Fluency	−0.87 (1.11)	−0.54 (1.31)	−1.16 (0.83)	0.13
Tower of London	−1.05 (2.00)	−0.89 (1.93)	−1.19 (2.09)	0.6

^a^ Mean (SD); n (%).

^b^ Kruskal-Wallis rank-sum test.

^c^ Fisher's exact test.

## Appendix

[Table table5-20552076221133758]

**Appendix 2. table5-20552076221133758:** Baseline demographics and assessments between BIDMC and Sangath-AIIMS Bhopal.

Variable	Overall, N = 40^a^	BIDMC, N = 11^a^	Sangath, N = 21^a^	p-value^b,c^
Age	30 (7)	31 (8)	29 (7)	0.7
Sex				0.071^c^
Female	22 (55%)	12 (71%)	10 (43%)	
Male	17 (42%)	4 (24%)	13 (57%)	
Non-binary	1 (2.5%)	1 (5.9%)	0 (0%)	
PHQ-9	16 (6)	14 (6)	17 (5)	0.053
GAD-7	12 (6)	10 (6)	13 (5)	0.070
WSS	9 (4)	5 (2)	12 (2)	<0.001
PSQI	9 (5)	12 (5)	6 (3)	<0.001
PANSS				
Positive	15 (5)	16 (5)	13 (5)	0.091
Negative	12 (5)	10 (3)	13 (5)	0.066
General	28 (7)	27 (4)	29 (8)	>0.9
Total	54 (13)	53 (7)	55 (16)	0.9
BACS				
Verbal Memory	−1.91 (1.37)	−1.42 (1.17)	−2.27 (1.41)	0.087
Digit Sequencing	−1.88 (1.55)	−0.97 (1.11)	−2.56 (1.50)	<0.001
Verbal Fluency	−1.76 (1.61)	−0.54 (1.31)	−2.67 (1.16)	<0.001
Tower of London	−1.85 (2.20)	−0.89 (1.93)	−2.56 (2.16)	0.003

^a^ Mean (SD) n (%).

^b^ Wilcoxon rank-sum test.

^c^ Fisher's exact test.

## Appendix

[Table table6-20552076221133758]

**Appendix 3. table6-20552076221133758:** Baseline demographics and assessments between NIMHANS and Sangath-AIIMS Bhopal.

Variable	Overall, N = 40^a^	NIMHANS, N = 18^a^	Sangath, N = 21^a^	p-value^b,c^
Age	31 (7)	32 (7)	29 (7)	0.14
Sex				0.8
Female	17 (42%)	8 (44%)	9 (41%)	
Male	23 (57%)	10 (56%)	13 (59%)	
Religion				0.7
Christian	1 (2.5%)	1 (5.6%)	0 (0%)	
Hindu	37 (92%)	16 (89%)	21 (95%)	
Muslim	2 (5.0%)	1 (5.6%)	1 (4.5%)	
PHQ-9	10 (8)	3 (3)	17 (5)	<0.001
GAD-7	7.4 (6.5)	1.5 (3.1)	12.2 (4.1)	<0.001
WSS	6.9 (5.6)	1.0 (1.1)	11.8 (1.9)	<0.001
PSQI	4.05 (2.49)	2.06 (1.70)	5.68 (1.74)	<0.001
PANSS				
Positive	11 (5)	8 (2)	13 (5)	<0.001
Negative	11 (5)	10 (3)	13 (5)	0.019
General	24 (8)	19 (3)	29 (8)	<0.001
Total	47 (15)	37 (5)	55 (16)	<0.001
BACS				
Verbal Memory	−2.32 (1.31)	−2.37 (1.23)	−2.27 (1.41)	0.8
Digit Sequencing	−2.01 (1.58)	−1.38 (1.47)	−2.56 (1.50)	0.029
Verbal Fluency	−1.97 (1.26)	−1.16 (0.83)	−2.67 (1.16)	<0.001
Tower of London	−1.92 (2.21)	−1.19 (2.09)	−2.56 (2.16)	0.012
Symbol Coding	−2.36 (1.39)	−1.82 (1.23)	−2.82 (1.38)	0.009
Token Motor Task	−2.28 (1.17)	−2.06 (1.03)	−2.48 (1.26)	0.2

^a^ Mean (SD); n (%).

^b^ Wilcoxon rank-sum test.

^c^ Pearson's Chi-squared test.

## Appendix

[Table table7-20552076221133758]

**Appendix 4. table7-20552076221133758:** Mean of baseline and monthly follow-up demographics and clinical assessments by study site and overall.

Variable	Overall, N = 53^a^	BIDMC, N = 13^a^	NIMHANS, N = 18^a^	Sangath-AIIMS Bhopal, N = 22^a^	p-value^b,c^
Age	30.74 (7.30)	30.85 (8.18)	32.33 (7.41)	29.36 (6.72)	0.3
Sex					0.12^c^
Female	26 (49%)	9 (69%)	8 (44%)	9 (41%)	
Male	26 (49%)	3 (23%)	10 (56%)	13 (59%)	
Non-binary	1 (1.9%)	1 (7.7%)	0 (0%)	0 (0%)	
Ethnicity					0.002
Hispanic or Latino	4 (7.5%)	4 (31%)	0 (0%)	0 (0%)	
NOT Hispanic or Latino	49 (92%)	9 (69%)	18 (100%)	22 (100%)	
Race					<0.001
African American	3 (5.7%)	3 (23%)	0 (0%)	0 (0%)	
Asian	40 (75%)	0 (0%)	18 (100%)	22 (100%)	
Multiracial or other	2 (3.8%)	2 (15%)	0 (0%)	0 (0%)	
White	8 (15%)	8 (62%)	0 (0%)	0 (0%)	
PHQ-9	11 (8)	13 (6)	3 (3)	17 (5)	<0.001
GAD-7	7.9 (6.4)	9.5 (6.0)	1.5 (3.1)	12.2 (4.1)	<0.001
WSS	6.4 (5.1)	4.6 (2.3)	1.0 (1.1)	11.8 (1.9)	<0.001
PSQI	5.5 (4.0)	9.9 (4.6)	2.1 (1.7)	5.7 (1.7)	<0.001
PANSS				
Positive	12.2 (5.0)	16.5 (4.2)	7.9 (1.9)	13.2 (4.6)	<0.001
Negative	10.80 (3.70)	9.85 (2.72)	8.75 (3.02)	13.05 (3.58)	<0.001
General	23.9 (6.3)	26.7 (2.3)	18.4 (3.5)	26.8 (6.8)	<0.001
Total	47 (12)	53 (6)	35 (6)	53 (12)	<0.001
BACS				
Verbal Memory	−2.01 (1.33)	−1.24 (1.13)	−2.25 (1.15)	−2.27 (1.45)	0.077
Digit Sequencing	−1.75 (1.54)	−0.88 (1.14)	−1.25 (1.36)	−2.68 (1.41)	0.001
Verbal Fluency	−1.67 (1.42)	−0.42 (1.38)	−1.15 (0.87)	−2.85 (0.80)	<0.001
Tower of London	−1.67 (2.20)	−0.56 (1.75)	−1.35 (2.14)	−2.58 (2.21)	0.004
Symbol Coding	−2.39 (1.43)	NA (NA)	−1.76 (1.29)	−2.90 (1.35)	0.005
Token Motor Task	−2.36 (1.16)	NA (NA)	−2.10 (1.08)	−2.57 (1.21)	0.2

^a^ Mean (SD); n (%).

^b^ Kruskal-Wallis rank-sum test.

^c^ Fisher's exact test.

## Appendix

[Table table8-20552076221133758]

**Appendix 6. table8-20552076221133758:** Mean activities completed per day for surveys and games, and per month for tips and relaxation activities, by study site and overall.

Characteristic	Overall, N = 53^a^	BIDMC, N = 13^a^	NIMHANS, N = 18^a^	Sangath-AIIMS Bhopal, N = 22^a^	p-value^b^
Surveys per month	178 (127)	127 (102)	236 (165)	159 (80)	0.089
Games per month	77 (97)	52 (42)	100 (145)	73 (63)	0.8
Tips per month	4.0 (10.7)	1.2 (2.1)	3.8 (14.8)	5.9 (9.6)	0.015
Breathe per month	5 (11)	6 (7)	5 (14)	5 (10)	0.025
Total activities	8.8 (7.0)	6.2 (4.7)	11.5 (9.8)	8.1 (4.4)	0.2
Games per day	2.58 (3.22)	1.74 (1.41)	3.35 (4.84)	2.44 (2.09)	0.8
Surveys per day	5.9 (4.2)	4.2 (3.4)	7.9 (5.5)	5.3 (2.7)	0.089

^a^ Mean (SD).

^b^ Kruskal-Wallis rank-sum test

## Appendix

[Fig fig7-20552076221133758][Fig fig8-20552076221133758]

## Appendix

[Fig fig9-20552076221133758]
